# Comparison of the nutrient content of children’s menu items at US restaurant chains, 2010–2014

**DOI:** 10.1186/s12937-015-0066-4

**Published:** 2015-08-15

**Authors:** Andrea L. Deierlein, Kay Peat, Luz Claudio

**Affiliations:** 1 Gustave Levy Place Department of Preventive Medicine, Icahn School of Medicine at Mount Sinai, 10029 NY New York, USA; 2 School of Public Health, City University of New York, NY New York, USA

## Abstract

**Objective:**

To determine changes in the nutritional content of children’s menu items at U.S. restaurant chains between 2010 and 2014.

**Methods:**

The sample consisted of 13 sit down and 16 fast-food restaurant chains ranked within the top 50 US chains in 2009. Nutritional information was accessed in June-July 2010 and 2014. Descriptive statistics were calculated for nutrient content of main dishes and side dishes, as well as for those items that were added, removed, or unchanged during the study period.

**Results:**

Nutrient content of main dishes did not change significantly between 2010 and 2014. Approximately one-third of main dishes at fast-food restaurant chains and half of main dishes at sit down restaurant chains exceeded the 2010 Dietary Guidelines for Americans recommended levels for sodium, fat, and saturated fat in 2014. Improvements in nutrient content were observed for side dishes. At sit down restaurant chains, added side dishes contained over 50 % less calories, fat, saturated fat, and sodium, and were more likely to contain fruits/vegetables compared to removed sides (*p* < 0.05 for all comparisons). Added side dishes at fast-food restaurant chains contained less saturated fat (*p* < 0.05).

**Conclusions:**

The majority of menu items, especially main dishes, available to children still contain high amounts of calories, fat, saturated fat, and sodium. Efforts must be made by the restaurant industry and policy makers to improve the nutritional content of children’s menu items at restaurant chains to align with the Dietary Guidelines for Americans. Additional efforts are necessary to help parents and children make informed choices when ordering at restaurant chains.

## Introduction

The food environment plays an influential role in shaping the diets of children and may contribute to the development of obesity and other adverse health outcomes [1, 2]. Foods purchased outside the home, specifically at restaurants, tend to be energy dense and have low nutritional value. A previous study evaluated nearly 3,500 children’s meal combinations offered at 34 of the top 50 restaurant chains in the United States (US), with respect to the National Restaurant Association’s KidsLiveWell standards, which provide specifications for calories, total fat, saturated fat, trans fat, sodium, sugar, and inclusion of food groups (i.e. vegetables and fruits) [3]. The study found that 91 % of the meals did not meet the KidsLiveWell standards and over a quarter of the restaurants did not have any meals that met these standards [3]. This is of concern because restaurant food and fast-food are common in children's diets, contributing approximately 84–191 kcal and 160–404 kcal to the mean daily intakes of children and adolescents, respectively, who reported being consumers [4]. A recent analysis of NHANES 2007–2010 showed that half of US children, ages 2–18 years, consumed fast-food and 10.5 % were high consumers, with more than 30 % of their daily calories coming from fast-food restaurants [5].

Over the past several years, there has been increasing media attention paid to the “healthfulness” of restaurant foods. Beginning in 2006, several municipalities implemented menu labeling laws and in March 2010, a federal menu labeling law (Section 4205 of the Patient Protection and Affordable Care Act) was instituted [6]. The law requires restaurant chains with more than 20 outlets in the US to display the number of calories contained in each item on the menu. Implementation of the law was originally expected in 2013 but was delayed so that it will be fully effective by December 2015. Though the law was mostly intended to assist consumers in making more informed decisions about meal selections, it is not clear whether restaurant chains consequently made changes to menu items to improve nutritional content [7]. In a study of changes in the nutrient content of adult entrees at sit-down and quick-service restaurant chains in the 18 months following jurisdiction-mandated menu labeling in King County, Washington, only very modest improvements were observed and levels of energy, saturated fat, and sodium remained excessive [8]. Few studies have examined longitudinal changes in children’s menu items offered at restaurant chains, revealing little difference in the nutrient content of menu items between 2008 and 2012 [9–11].

There are currently no studies, to our knowledge, that evaluate the most recent changes in children’s menus offered at both sit down and fast-food restaurant chains. The purpose of this study was to determine longitudinal changes in the nutrient and fruit/vegetable content of main and side dishes available on children’s menus at popular US restaurant chains between 2010 and 2014. This time period approximately captures the introduction and pending implementation of the federal menu labeling law.

## Methods

The sample consisted of restaurant chains ranked within the top 50 chains in the US in 2009 [12]. Rankings were based on restaurant chains’ total sales in 2008. Restaurant chains that offered only buffets, pizza, or convenience/snack options (ice cream, pastries, coffee, and other convenience items) were excluded (*n* = 11). Since the objective of this study was to describe the nutrient profiles of children’s menus items, restaurant chains that only provided one menu (presumably an adult menu) were excluded (*n* = 4). Restaurant chains that did not provide nutritional information for the menus were excluded (*n* = 6). Lastly, one fast-food restaurant chain no longer offered a separate children’s menu at follow-up and was excluded from analyses. The remaining 28 restaurant chains represented over $177 billion in total sales for 2008 (~73 % of sales for all chains ranked within the top 50), with fast-food restaurant chains accounting for approximately $150 billion of the sales (~85 % of sales for restaurant chains within the study sample). Restaurant chains were then classified as sit-down chains (*n* = 13) and fast-food chains (*n* = 15). Sit down restaurant chains were defined as those with wait-staff service and fast-food restaurant chains were defined as those offering self-service or carry-out without wait-staff service [13].

Menus and nutritional data were collected from each restaurant chain’s website in June-July 2010 and at follow-up in June-July 2014. Nutritional data were entered for main dishes and side dishes “as offered” or “as served” by each chain. Main dishes that were served with specific side dishes (i.e. hamburger with French fries or macaroni and cheese with a fruit cup) or specific dressings/sauces (i.e. baby carrots with ranch dressing) within a given restaurant chain were included in analyses as single items. Main dishes or side dishes that were offered both with and without a specific accompaniment (i.e. hamburger with a choice of a side or apple slices with or without caramel sauce) within a given restaurant chain were included in analyses as separate items. Salads were included with no dressing, unless served with a specific dressing (i.e. Caesar salad). In the case of “build your own” sandwiches, average nutritional information for the base sandwich (i.e. turkey with the average nutritional information for the bread choices and cheese choices) offered on the menu was used without additional toppings and condiment options. The following menu items were excluded from analysis: beverages; dessert items (i.e. cookies and sweets); additional dressings/sauces; special diet; seasonal or region-specific items; and meals specifically only served at lunch or breakfast. There was one fast-food restaurant chain that offered side dishes (bagged chips) without posted nutritional information in both years. These side dishes were excluded from analyses.

### Statistical analysis

Nutritional data were entered into an Excel database and Stata 13.0 (College Station, TX) was used for statistical analyses. Descriptive statistics (means and standard deviations, SD) were computed for total calories, fat (g), percent calories from fat, total saturated fat (g), percent calories from saturated fat, fiber (g), and sodium (mg). Five sit down restaurant chains did not provide complete information for all of the selected nutrient variables; therefore, among sit down restaurants, the full sample size is not available for saturated fat, sodium, and fiber. The number and percent of main and side dishes containing fruits and/or vegetables (as described on the menu) were also calculated. For example, main dishes of vegetable stir fry and chicken tenders with a fruit cup were considered to contain fruits/vegetables, while side dishes of celery sticks or apple slices were also considered to contain fruits/vegetables. Differences in means of nutrient content or percent of dishes containing fruit/vegetable between 2010 and 2014 at fast-food and sit down restaurant chains were evaluated using t-tests and chi-square tests (*p* < 0.05). Any nutritional data that were reported as “<1” on the restaurant chain’s website, such as a food item containing <1 g of fat, was entered as 1 in the database. Values for sodium represented sodium that was either naturally occurring in the food or was added during processing or preparation, not sodium from table salt. The majority of restaurant chains did not provide information regarding the percentages of calories from fats; therefore, percentages were calculated assuming 9 cal per gram of fat and saturated fat. The proportion (%) of main dishes and side dishes not meeting the 2010 Dietary Guidelines for Americans [14] for percent of calories from fat (<=35 % for 4–18 year olds) and saturated fat (<10 %) as well as sodium (mg) were calculated. According to the Guidelines, the recommendation for sodium for children is <2300 mg/day (tolerable upper intake level). Based on this recommendation, we established <500 mg for an entrée and <200 mg for a side as the recommended level per meal (i.e. <700 mg), which is slightly less than a third of the daily recommendation. Items containing fried vegetables, such as fried potatoes (French fries) and onion rings, or items containing fruit flavorings, such as strawberry yogurt, were considered as not containing fruits/vegetables.

Changes in children’s menus between 2010 and 2014 were also determined. Main dishes and side dishes were categorized as “Added”, “Removed”, or “Unchanged” as adapted from a previous study [11]. Added items were those that were not offered on the 2010 menu but offered on the 2014 menu; removed items were offered on the 2010 menu but not offered on the 2014 menu; and unchanged items were offered on both the 2010 and 2014 menus. Items with name changes only, with no changes in preparation method (as described on the menu), were considered unchanged. For example, if a 2010 menu offered fried chicken strips and the 2014 menu offered chicken tenders, these items were considered to be *unchanged* because both items are prepared similarly. However, if the 2014 menu offered grilled chicken strips, then fried chicken strips were considered to be *removed* and grilled chicken strips were considered to be *added* because the preparation method changed. Mean nutrient and fruit/vegetable content of added, removed, and unchanged menu items were compared between 2010 and 2014 using t-tests (*p* < 0.05).

## Results

### Main dishes

Descriptive statistics for nutrient and fruit/vegetable content of main dishes available on children’s menus, as well as removed and added main dishes, at fast-food and sit down restaurant chains are shown in Table 1. At both fast-food and sit down restaurant chains, the average nutrient content of main dishes did not substantially change between 2010 and 2014. The only exception being that main dishes at fast-food restaurant chains had a lower mean percentage of calories from saturated fat in 2014 compared to 2010 (*p* < 0.05). Similarly, no statistically significant changes in average nutrient content were observed for removed and added main dishes at fast-food or sit down restaurant chains (bottom half of Table 1). The nutrient contents of unchanged main dishes (*n* = 45 at fast-food restaurant chains and *n* = 83 at sit down restaurant chains) were nearly identical between 2010 and 2014 (data not shown).

**Table 1:** Descriptive statistics of nutrient content of main dishes offered on children's menus, as well as removed and added main dishes, at popular US fast-food and sit down restaurant chains, 2010 and 2014

Nutrient Characteristic	Fast-Food				Sit down			
	2010		2014		2010		2014	
	N	Mean (SD)	N	Mean (SD)	N	Mean (SD)	N	Mean (SD)
Calories	66	268.3 (101.5)	68	277.9 (96.7)	99	381.0 (174.7)	112	403.4 (192.6)
Fat (g)	66	12.1 (6.5)	68	12.3 (6.0)	99	18.7 (13.0)	112	19.5 (12.8)
% of Calories from Fat	66	39.5 (12.1)	68	39.0 (12.0)	99	40.4 (16.8)	112	40.8 (15.7)
Saturated Fat (g)	66	4.5 (3.1)	68	4.0 (2.7)	75	6.4 (5.8)	85	6.8 (5.6)
% of Calories from Saturated Fat	66	14.4 (7.7)	68	12.0 (5.4)*	75	13.3 (8.7)	85	14.5 (9.3)
Sodium (mg)	66	664.7 (245.3)	68	663.8 (267.0)	99	846.9 (459.2)	112	937.5 (502.7)
Fiber (g)	66	1.5 (1.0)	68	1.7 (1.2)	85	2.1 (2.0)	92	2.0 (1.8)
Fruits and Vegetables, n (%)	66	1 (1.5)	68	1 (1.5)	99	8 (8.1)	112	6 (5.4)
	Removed		Added		Removed		Added	
	N	Mean (SD)	N	Mean (SD)	N	Mean (SD)	N	Mean (SD)
Calories	21	265.2 (111.8)	24	280.4 (91.4)	16	447.9 (191.1)	29	491.8 (229.5)
Fat (g)	21	13.1 (7.0)	24	12.9 (5.8)	16	23.3 (14.6)	29	25.6 (14.3)
% of Calories from Fat	21	43.8 (11.5)	24	40.6 (10.6)	16	43.8 (16.0)	29	45.4 (14.8)
Saturated Fat (g)	21	4.7 (3.5)	24	3.8 (2.3)	12	8.7 (8.5)	23	8.5 (6.1)
% of Calories from Saturated Fat	21	14.8 (8.1)	24	11.5 (4.6)	12	15.4 (10.4)	23	16.1 (8.3)
Sodium (mg)	21	622.4 (253.8)	24	650.8 (276.8)	16	870.8 (450.4)	29	1108.1 (599.2)
Fiber (g)	21	1.2 (0.9)	24	1.8 (1.2)	16	2.3 (1.8)	26	2.3 (1.8)
Fruits and Vegetables, n (%)	21	0 (0)	24	0 (0)	16	3 (18.8)	29	1 (3.5)

*P < 0.05 t-tests or chi square tests comparing nutrient contents of main dishes between 2010 and 2014 (top half of table), as well as removed and added main dishes (bottom half of table), at each restaurant chain type

**Fig. 1:**
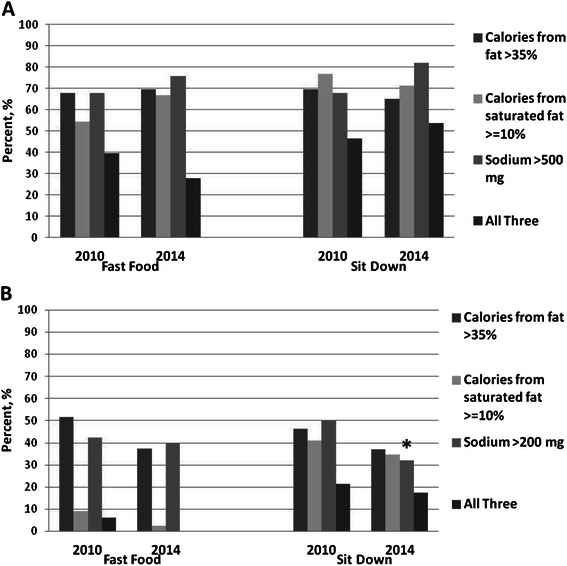
Percent of main dishes (**a**)^a^ and side dishes (**b**)^b^ exceeding recommendations for percent of calories from fat, percent of calories from saturated fat, and sodium (mg), and for all three recommendations^c^ at fast-food and sit down restaurant chains, 2010–2014. ^a^Sample sizes for main dishes at fast food restaurants: n=66 in 2010 and n=68 in 2014 .Sample sizes for main dishes at sit down restaurants: n=99 for fat and sodium, n=75 for saturated fat in 2010; n=112 for fat and sodium, n=85 for saturated fat in 2014. ^b^Sample sizes for side dishes at fast-food restaurants: n=33 in 2010 and n=40 in 2014. Sample sizes for side dishes at sit down restaurants: n=56 for fat and sodium, n=47 for saturated fat in 2010; n=81 for fat and sodium, n=70 for saturated fat in 2014. ^c^There were no side dishes at fast food restaurant chains that exceeded all three recommendations in 2014. *p<0.05 comparing percent of side dishes exceeding recommendations for sodium content in 2010 and 2014

The majority of main dishes at fast-food and sit down restaurant chains exceeded recommendations for percent of calories from fat (>35 %), percent of calories from saturated fat (> = 10 %), and sodium (>500 mg), with no statistically significant changes between 2010 and 2014 (Fig. 1). In 2014, the proportion of main dishes exceeding recommendations for sodium and calories from fat and saturated fat at fast-food and sit down restaurant chains ranged from 65–82 %, and the proportion exceeding all three recommendations (fat, saturated fat, *and* sodium) was approximately 28 and 54 % at fast-food and sit down restaurant chains, respectively.

### Side dishes

Descriptive statistics for nutrient and fruit/vegetable content of side dishes available on children’s menus, as well as removed and added side dishes, at fast-food and sit down restaurants are shown in Table 2. The average nutrient content of side dishes offered at fast-food restaurant chains was similar between 2010 and 2014. Statistically significant differences were found for side dishes at sit down restaurant chains between 2010 and 2014. On average, side dishes offered in 2014 contained approximately 50 fewer calories (*p* = 0.03) and 125 mg less sodium (*p* = 0.03). A higher percentage of side dishes contained fruits/vegetables (78.6 % in 2014 versus 66.1 % in 2010), though this difference was not statistically significant.

**Table 2:** Descriptive statistics of nutrient content of side dishes offered on children's menus, as well as removed and added side dishes, at popular US fast-food and sit down restaurant chains, 2010 and 2014

Nutrient Characteristic	Fast-Food				Sit Down			
	2010		2014		2010		2014	
	N	Mean (SD)	N	Mean (SD)	N	Mean (SD)	N	Mean (SD)
Calories	33	150.7 (86.7)	40	127.1 (88.4)	56	180.2 (135.1)	84	134.3 (116.7)*
Fat (g)	33	6.0 (6.0)	40	4.5 (5.4)	56	7.7 (8.6)	81	5.4 (7.6)
% of Calories from Fat	33	26.3 (22.4)	40	19.8 (21.6)	56	28.5 (25.8)	81	23.4 (25.2)
Saturated Fat (g)	33	1.1 (1.1)	40	0.7 (0.9)	47	2.3 (3.1)	70	1.6 (2.3)
% of Calories from Saturated Fat	33	5.0 (5.8)	40	3.0 (3.6)	47	8.0 (9.7)	70	6.7 (11.4)
Sodium (mg)	33	248.2 (252.6)	40	205.4 (223.1)	56	325.5 (418.2)	81	197.2 (263.8)*
Fiber (g)	33	2.1 (1.6)	40	1.9 (1.0)	46	2.7 (2.2)	69	2.1 (1.5)
Fruits and Vegetables, n (%)	33	16 (48.5)	40	19 (47.5)	56	37 (66.1)	84	66 (78.6)
	Removed		Added		Removed		Added	
	N	Mean (SD)	N	Mean (SD)	N	Mean (SD)	N	Mean (SD)
Calories	11	134.5 (76.9)	18	89.4 (66.0)	18	230.0 (157.9)	46	111.2 (85.8)***
Fat (g)	11	4.0 (5.7	18	1.8 (3.4)	18	11.5 (9.6)	44	5.1 (7.3)**
% of Calories from Fat	11	19.8 (23.0)	18	9.7 (17.1)	18	38.7 (27.2)	44	26.0 (27.8)
Saturated Fat (g)	11	0.68 (0.98)	18	0.17 (0.34)^*^	15	4.1 (4.1)	39	1.5 (2.4)**
% of Calories from Saturated Fat	11	4.6 (8.2)	18	0.9 (1.7)	15	12.6 (10.7)	44	6.0 (8.8)*
Sodium (mg)	11	205.5 (281.6)	18	132.5 (174.4)	18	581.3 (571.5)	44	216.7 (275.9)***
Fiber (g)	11	2.2 (2.5)	18	1.7 (1.1)	12	3.5 (3.2)	37	1.9 (1.1)**
Fruits and Vegetables, n (%)	11	8 (72.7)	18	11 (61.1)	18	12 (66.7)	46	41 (89.1)*

*P < 0.05 **P < 0.01 ***P < 0.001 t-tests or chi square tests comparing nutrient contents between 2010 and 2014, as well as removed and added side dishes, at each restaurant chain type

Differences in nutrient content were also observed between removed and added side dishes (bottom half of Table 2). Added side dishes at fast-food restaurant chains contained less saturated fat than removed side dishes (*p* = 0.05). The most noticeable differences were observed for side dishes at sit down restaurant chains: compared to removed side dishes, added side dishes contained over 50 % less calories, fat, saturated fat, and sodium and were more likely to contain fruits/vegetables (*p* < 0.05 for all comparisons). They also contained less fiber (3.5 g in removed items versus 1.9 g in added items, *p* = 0.01). The nutrient contents of unchanged side dishes (*n* = 22 at fast-food restaurant chains and *n* = 38 at sit down restaurant chains) were nearly identical between 2010 and 2014 (data not shown).

Overall, the proportion of side dishes at fast-food and sit down restaurant chains that exceeded recommendations for percent of calories from fat (>35 %), percent of calories from saturated fat (<=10 %), and sodium (<200 mg) decreased between 2010 and 2014. However, only the difference in saturated fat content at sit down restaurant chains was statistically significant; in 2010 approximately 50 % contained at least 10 % of calories from saturated fat compared to 32 % in 2014 (*p* = 0.04) (Fig. 1).

## Discussion

The purpose of this study was to determine changes in the nutritional content of main dishes and side dishes offered on children’s menus at popular U.S. sit down and fast-food restaurant chains between 2010 and 2014. The nutrient content of main dishes offered at sit down and fast-food restaurant chains did not appreciably change and the nutrient content of removed and added main dishes was similar during the study time period. The majority of main dishes remained high in fat, saturated fat, and sodium. Improvements were observed, however, for side dishes, which tended to be lower in calories, fat, saturated fat, and sodium in 2014 compared to 2010. Statistically significant differences were observed for side dishes offered at sit down restaurants; compared to removed side dishes, added side dishes contained over 50 % less calories, fat, saturated fat, and sodium and were more likely to contain fruits/vegetables (*p* < 0.05 for all comparisons).

Recent studies, using nutrition data from 2010, report poor nutritional content of meals offered on children’s menus at restaurant chains [15–18]. In a study of 245 restaurant chains, only 11 % of main dishes and 33 % of sides offered on children’s menus met the KidsLiveWell standards (created by the National Restaurant Association) [15]. When comparing main dishes to criteria based on the US Department of Agriculture Recommended Dietary Allowance [14] of energy, fat, saturated fat, and sodium, only 8 % of main dishes met all four criteria for 4–8 year olds (<533 cal, <=35 % energy from fat, <=10 % energy from saturated fat, and <633 mg sodium) and 11 % of main dishes met all criteria for 9–13 year olds (<633 cal, <=35 % energy from fat, <=10 % energy from saturated fat, and <733 mg sodium) [15]. In the present study, main dishes and side dishes at both types of restaurant chains consistently contained high amounts of fat, saturated fat, and sodium and low amounts of fiber and fruits/vegetables, though values tended to be higher at sit down restaurant chains. This observation has been reported previously and is likely due to larger portion sizes served at sit down compared to fast-food restaurant chains [19].

Few studies have examined recent changes in menus targeted to children. One study determined changes in meal combinations (main dishes, sides, and beverages) offered on children’s menus at 34 US restaurant chains, 2008–2012 [9]. The percentage of meals that met nutrition standards for calories (<=430 kcal) and sodium (<=770 mg) approximately doubled, from 7 to 14 % and 15 to 34 %, respectively, during the study period. Additionally, a higher percentage of restaurant chains offered meal combinations with some type of fruit or vegetable, including juices (69 % in 2008 versus 72 % in 2012). However, the percentage of meals that met standards for fat (<=35 % of calories) remained the same, at 54 %, and the percentage that met standards for saturated plus trans fat (<=10 % of calories) decreased from 54 % in 2008 to 45 % in 2012. Those results are consistent with the results from the present study and suggest that, although there was no difference in the average nutrient content of main dishes, it is likely the overall nutrient content of many meals (main dishes combined with side dishes) would improve due to the observed decreases in calories, fat, and sodium and increases in fruit/vegetable content of side dishes.

Other studies examined longitudinal changes with respect to jurisdictional and federal menu labeling policies [10, 11]. In an analysis of fast-food restaurant chains with (cases, *n* = 5) and without (controls, *n* = 4) jurisdiction-mandated calorie-labeling on menus, 2005–2011, there was little improvement in the healthiness of children’s main dishes during the study period. Healthy entrées were defined as containing < =25 % of the dietary reference value of calories, saturated fat, sodium, cholesterol, and fiber. Only one case restaurant and no control restaurants offered children’s main dishes meeting 4 of the 5 nutritional criteria [10]. Another study reported no changes in energy or sodium content of children’s main dishes at 109 restaurant chains between 2010 and 2011. However, after adjusting for several restaurant characteristics including restaurant size, type of meal (i.e. breakfast, lunch, or dinner), and cuisine, there was an estimated average decrease of 40 cal in main dishes at fast-food restaurant chains (*n* = 21), with a difference of −57 cal in added versus removed main dishes [11].

It is difficult to make direct comparisons in the results across studies due to differences in the time period that data were collected, types and number of restaurant chains included, the unit of analysis (such as combination meals or individual items, inclusion of beverages or breakfast and lunch items), and statistical methodology. Our study, as well as others, is limited to the accuracy and validity of nutrition information posted on restaurants’ websites, which may be under-reported [20] and likely underestimates the average nutrient values. We are also unable to account for any additions made after food preparation or beverages consumed with the meals, which would likely account for substantial increases in calories, macronutrients, and sodium. We cannot comment on parent and child purchasing behaviors, which may not be influenced by nutrition menu labeling [21]. These limitations suggest that the reported nutrient content of meals is lower than what is actually being consumed by children. Lastly, though the federal labeling law was introduced in 2010 with support from the National Restaurant Association, its effective date was delayed until December 2015. Therefore, this study only approximately captures the time period from introduction to the pending implementation of the law. Although our results suggest that some improvements in the nutrient content of children’s menu items were made during this time, we cannot comment on any additional changes to menu offerings and/or nutritional content during the time period leading up to full implementation of the law.

## Conclusions

Taken collectively, the results show that small changes have been made in the past several years to improve the average nutrient and fruit/vegetable content of meals (main dishes with side dishes) offered to children at restaurant chains. These changes do not seem to be due to a reformulating of the nutrient content of existing menu items but rather to replacement of some menu items with healthier options, specifically side dishes. The evidence suggests that little has been done to improve the nutrient and fruit/vegetable content of main dishes. Therefore, although healthier options are available and the average nutrient content of meals may be improved in some cases, the majority of the selections available to children still contain high amounts of calories, fat, saturated fat, and sodium. Future studies are needed to determine whether full implementation of the menu-labeling laws, as well as other social pressures, result in further improvements in nutrient content of children’s meals at restaurant chains. Continuing efforts made by the restaurant industry and policy makers to increase the nutritional value of children’s meals at chain restaurants, especially main dishes, and to help parents and children make informed choices when ordering at these restaurants are necessary.

## References

[1] Hoyt LT, Kushi LH, Leung CW, Nickleach DC, Adler N, Laraia BA, et al. Neighborhood influences on girls' obesity risk across the transition to adolescence. Pediatrics. 2014;134. 10.1542/peds.2014-1286 PMC4533282 25311606

[2] Osei-Assibey G, Dick S, Macdiarmid J, Semple S, Reilly JJ, Ellaway A (2012). The influence of the food environment on overweight and obesity in young children: a systematic review. BMJ Open.

[3] Batada A, Bruening M, Marchlewicz EH, Story M, Wootan MG (2012). Poor nutrition on the menu: children's meals at America's top chain restaurants. Child Obes.

[4] Powell LM, Nguyen BT (2013). Fast-food and full-service restaurant consumption among children and adolescents: effect on energy, beverage, and nutrient intake. JAMA Pediatr.

[5] Poti JM, Duffey KJ, Popkin BM (2014). The association of fast food consumption with poor dietary outcomes and obesity among children: is it the fast food or the remainder of the diet?. Am J Clin Nutr.

[6] Patient Protection and Affordable Care Act. Public Law 111 e148, sec. 4205-nutrition labeling of standard menu items at chain restaurants. March, 2010; http://www.gpo.gov/fdsys/pkg/FR-2010-08-25/pdf/2010-21065.pdf Accessed July 1, 2014.

[7] Stein K (2010). A national approach to restaurant menu labeling: the Patient Protection and Affordable Health Care Act, Section 4205. J Am Diet Assoc.

[8] Bruemmer B, Krieger J, Saelens BE, Chan N. Energy, saturated fat, and sodium were lower in entrées at chain restaurants at 18 months compared with 6 months following the implementation of mandatory menu labeling regulation in King County, Washington. J Acad Nutr Diet. 2012;112:1169–76. 10.1016/j.jand.2012.04.019 22704898

[9] Batada A, Flewelling L, Goode A, Wootan MG. Kids' Meals II: Obesity and Poor Nutrition on the Menu. March, 2013. http://cspinet.org/new/pdf/cspi-kids-meals-2013.pdf Accessed July 1, 2014.

[10] Namba A, Auchincloss A, Leonberg BL, Wootan MG (2013). Exploratory analysis of fast-food chain restaurant menus before and after implementation of local calorie-labeling policies, 2005–2011. Prev Chronic Dis.

[11] Wu HW, Sturm R (2014). Changes in the energy and sodium content of main entrees in US chain restaurants from 2010 to 2011. J Acad Nutr Diet.

[12] Restaurants & Institutions Magazine. 2009 Top 400 restaurant chains. http://www.rimag.com/article/372414-R_I_2009_Top_400_Restaurants_Chains.phpUpdated July 15, 2009.Accessed June 1, 2010.

[13] Biing-Hwan L, Frazao E. Nutritional quality of foods at and away from home. Food review. US Government Printing Office, Food and Rural Economics Division, Economics Research Service, USDA. 1997:33-40.

[14] U.S.Department of Agriculture and U.S. (2010). Department of Health and Human Services. Dietary guidelines for Americans, 2010.

[15] Wu HW, Sturm R (2013). What's on the menu? A review of the energy and nutritional content of US chain restaurant menus. Public Health Nutr.

[16] Kirkpatrick SI, Reedy J, Kahle LL, Harris JL, Ohri-Vachaspati P, Krebs-Smith J (2013). Fast-food menu offerings vary in dietary quality, but are consistently poor. Public Health Nutr.

[17] Hobin E, White C, Li Y, Chiu M, O'Brien MF, Hammond D (2014). Nutritional quality of food items on fast-food ‘kids’ menus’: comparisons across countries and companies. Public Health Nutr.

[18] Wellard L, Glasson C, Chapman K (2012). Fries or a fruit bag? Investigating the nutritional composition of fast food children’s meals. Appetite.

[19] Serrano EL, Jedda VB (2009). Comparison of fast-food and non-fast-food children's menu items. J Nutr Educ Behav.

[20] Urban LE, McCrory MA, Dallal GE, Das SK, Saltzman E, Weber JL (2011). Accuracy of stated energy contents of restaurant foods. JAMA.

[21] Dodds P, Wofenden L, Chapman K, Wellard L, Hughes C, Wiggers J (2014). The effect of energy and traffic light labeling on parent and child fast food selection: a randomised controlled trial. Appetite.

